# Geographical variation and genetic diversity of *Parashorea chinensis* germplasm resources

**DOI:** 10.3389/fpls.2024.1452521

**Published:** 2024-10-16

**Authors:** Yuanyuan Xu, Shinan Liu, Patrick M. Finnegan, Fang Liu, Izhar Ali, Haidong Zhang, Mei Yang

**Affiliations:** ^1^ Key Laboratory of National Forestry and Grassland Administration on Cultivation of Fast-Growing Timber in Central South China, College of Forestry, Guangxi University, Nanning, China; ^2^ Guangxi Colleges and Universities Key Laboratory for Cultivation and Utilization of Subtropical Forest Plantation, College of Forestry, Guangxi University, Nanning, China; ^3^ School of Biological Sciences, University of Western Australia, Perth, WA, Australia; ^4^ Nanning Arboretum, Guangxi Zhuang Autonomous Region, Nanning, China

**Keywords:** *Parashorea chinensis*, phenotype, genetic diversity, ISSR, environmental factor

## Abstract

**Introduction:**

*Parashorea chinensis* is a rare monodominant species in southwest China known for its production of high-quality timber, is facing decline due to its narrow distribution, human interference and habitat destruction. However, there are no reports on genetic diversity and geographical variation of phenotypic traits of *P. chinensis*.

**Methods:**

In this study, phenotypic characters and genetic diversity of 15 germplasms resources from five provenances in southwest China were investigated, and their relationships with geographical and environmental factors was discussed.

**Results:**

Our results revealed a rich phenotypic diversity among the germplasms, with variation coefficients ranging from 3.63% to 45.49%. Among the studied germplasms, NP03 from Napo and ML02 from Mengla region exhibited superior phenotypic traits. Notably, NP03 also demonstrated the highest genetic diversity. Genetic differentiation analyses including genetic differentiation coefficient (0.6264) and gene flow (0.3736) illustrated that genetic variation was most prevalent among populations. Furthermore, redundancy analysis showed that temperature related factors (maximum air temperature, annual mean temperature and minimum air temperature) significantly affected phenotypic variation. Similarly, altitude, longitude, latitude, annual mean precipitation and the minimum air temperature significantly impacted the level of genetic diversity. The molecular variation of the natural population of *P. chinensis* followed a certain geographical pattern.

**Discussion:**

Our finding indicated abundant phenotypic variation among *P. chinensis* germplasms. However, populations exhibited low levels of genetic diversity alongside high genetic differentiation, potentially contributing to the species' rarity. Based on our results, NP03 and ML02 germplasm could be used as the parents for breeding superior germplasm of *P. chinensis*. Overall, this study provides valuable insights into germplasm diversity and conservation, genetic improvement, and utilization of *P. chinensis*.

## Introduction

*Parashorea chinensis* Wang Hsie is a tree species belonging to the family Dipterocarpaceae, mainly distributed in the valley’s southern Asian landform (Li et al., 2005; Zhu and Sun, 2019). It is also recognized as an iconic species in the tropical rainforests of China (Han et al., 2020). In China, it is mainly distributed in Mengla, Hekou, and Maguan counties in Yunnan Province and Napo and Tianyang counties in Guangxi Province. As a precious hardwood tree species, it has been utilized in construction, decoration and furniture making due to its tall and straight trunk, high timer yield, strong decay resistance and beautiful decorative pattern. However, this extensive historical utilization has significantly reduced tree resources through felling and utilization. Meanwhile, the survival of the tree species is threatened by its limited distribution and destruction of its primary forest habitat (Han et al., 2020). Additionally, the species faces challenges, including a single population genetic structure, high inbreeding within the population, the short lifespan of recalcitrant seeds and the difficulty of natural regeneration. These factors may seriously restrict the expansion of the population range and individual numbers due to changes in climate and native site conditions caused by human activities (Dai et al., 2017). It has been listed as a Class I endangered plant in Chin, and the International Union for Conservation of Nature and Natural Resources (UICN) (Dai et al., 2017; Li et al., 2004), which helps to raise awareness for the conservation and restoration of the tree population. Therefore, it is urgent to protect the natural forest genetic resources of *P. chinensis* to prevent the loss of its valuable genes and genetic diversity. Currently, there is a growing research interest aimed at expanding the *P. chinensis* population for conservation and cultivation, with a predominant focus on natural forest protection (Liu et al., 2018), seedling cultivation (Li et al., 2020; Wang et al., 2022) and plantation afforestation on non-native land (Han et al., 2020; Li et al., 2022; Ullah et al., 2024; Li et al., 2024). However, there are no reports on genetic diversity and geographical variation of phenotypic traits of *P. chinensis*.

Genetic diversity plays a crucial role in conservation efforts related to population management (Andayani et al., 2001). Phylogenetic reconstruction facilitates the discovery of greater plant diversity, aiding biologists in selecting areas or species to prioritize in conservation endeavors (Patel et al., 2015). A genetic marker suitable for diversity studies has a high degree of variability and can create multi-locus information from the studied genome (Al Salameen et al., 2018; Anne, 2006). Inter-simple sequence repeats (ISSR) analysis offers several advantages, including stable amplification, strong detection capability and high resolution (Sabir et al., 2014; Zietkiewicz et al., 1994), making it widely utilized in plant germplasm identification, genetic diversity analysis and genetic relationship analysis (Godwin et al., 1997; Kostas et al., 2021).

ISSR analysis has been extensively employed to study the genetic diversity of various plants, including herbaceous species like *Vigna unguiculata* (L.) Walp (Ghalmi et al., 2010), *Sesamum indicum* L (Woldesenbet et al., 2015), *Amaranthus* L (Singh et al., 2013), *Coix lacrymajobi* L (Fu et al., 2019), *Brassica rapa* subsp. rapa (Küçük et al., 2024), and *Mentha* L (Çelik et al., 2024), as well as woody plants like *Aquilaria sinensis* (lour.) gilg (Zou et al., 2012), *Prunus* L (Wu et al., 2018), *Ilex aquifolium* ‘Agrifoglio Commune’ (Tsaktsira et al., 2021), *Sapindus* L (Sun et al., 2019), and *Prunus spinosa* L (Gülay et al., 2023). Despite the numerous advantages of molecular marker technology for genetic diversity analysis, phenotypic traits are readily in the field. Phenotypic variation represents the most direct manifestation of plant genetic diversity, reflecting genotypic adaptation to the environment (Pigliucci and Kolodynska, 2006). Plant phenotypic traits are mainly controlled by heredity, and due to adaptation to different environmental conditions, their phenotypic traits have differentiation and variation, such as *Sapindus* L (Sun et al., 2017), *Liquidambar formosana* Hance (He et al., 2019), and *Phoebe bourne* (Hemsl.) Yen C. Yang (Tan et al., 2019). Moreover, plant phenotypic variation often correlates to environmental factors such as precipitation, altitude, and temperature in distribution locations (Rawat and Bakshi, 2011). Studying the relationship between environmental factors and genetic variation within *P. chinensis* populations, as well as understanding the patterns of geographical variation, holds significant theoretical importance. In this study, we collected 15 germplasms of *P. chinensis* from the main distribution sites in Guangxi and Yunnan provinces, China. Utilizing ISSR analysis, we investigated genetic diversity variation within *P. chinensis* population and analyzed the correlation between the genetic diversity and the environmental factors at the distribution locations. Our aim was to comprehend and predict the patterns of geographical variation within the *P. chinensis* population, providing fundamental data and a scientific basis for correct and rational utilization and protection of *P. chinensis* germplasm resources.

## Materials and methods

### Plant materials

**Figure 1:**
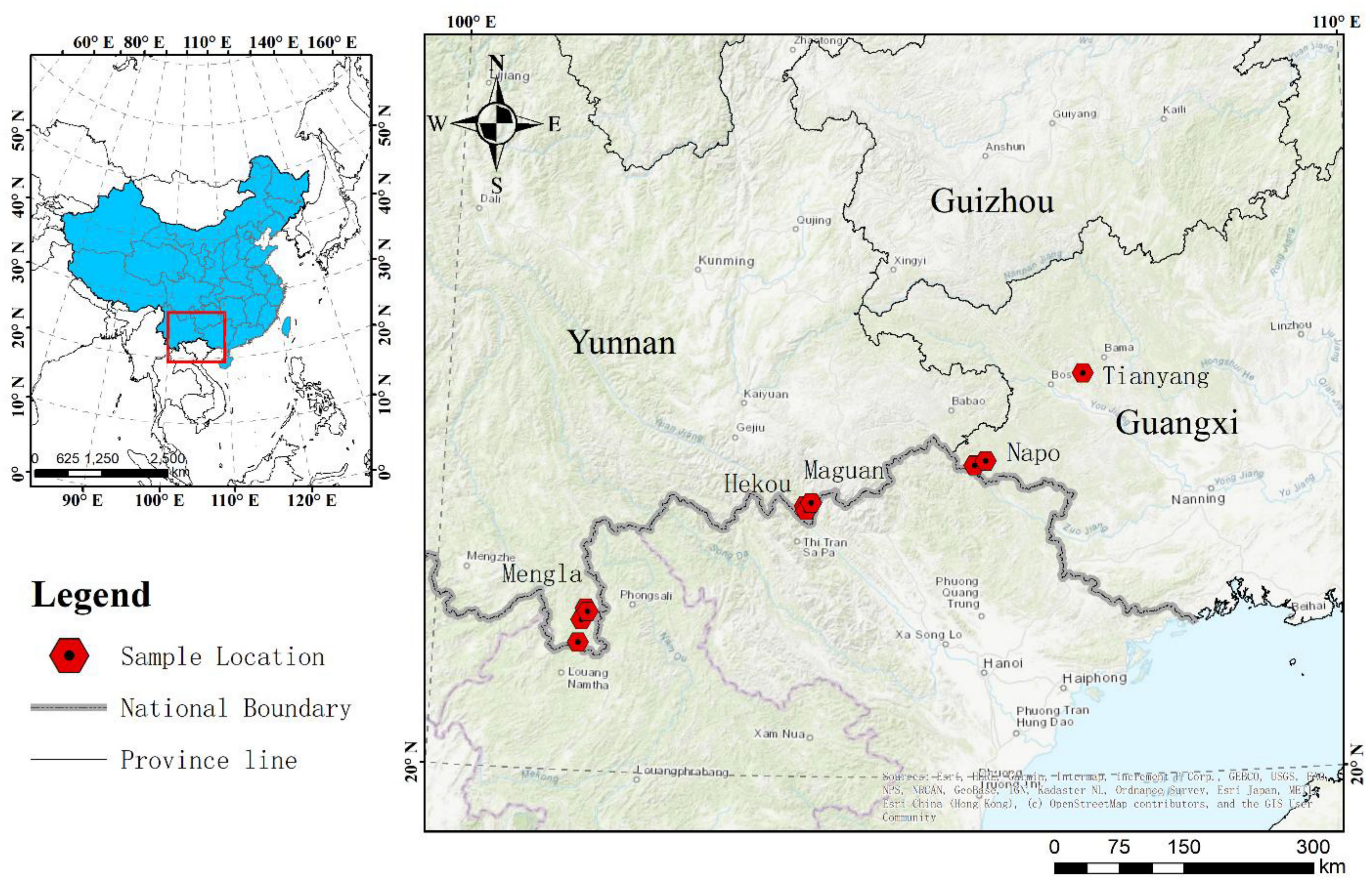
Location of collected samples.

Mature seeds of *P. chinensis* were collected from populations at 15 distinct localities covering most of its distribution area in China in July 2014. The plant materials were formally identified by Lecturer Rongyan Deng, and there are no conflicts of interest or legal issues. Samples were collected from Napo (NP) and Tianyang (TY) counties of Guangxi province, and from Hekou (HK), Maguan (MG) and Mengla (ML) counties of Yunnan province (**Figure 1**). The location and geographic descriptions of the collections are given in **Supplementary Table S1**. Seeds were transported to a nursery at the Nanning Arboretum, Guangxi, China (107°45′E, 22°13′N), as soon as possible after collection to prevent them from germinating before being embedded in seedling sand bed. The voucher specimens for seeds of NP, TY, HK, MG and ML are reserved in the rare species seed bank at College of Forestry, Guangxi University.

*P. chinensis* seed was sown in a sand bed to germinate and grow until the first true leaves reached maturity. Seedlings were transplanted to pots (one seedling per pot) measuring 210 mm diameter ×170 mm deep containing yellow soil. Watering was done as necessary to maintain 60% to 70% field water-holding capacity. Seedlings were grown under 50% light-exclusion shade net. Weed control was carried out regularly when required. 100 g compound fertilizer (N:P:K =15:6:9) per pot was applied in April, 2015 and 2016. A completely randomized design (CRD) was used, with three biological replicates per germplasm and 30 seedlings per replicate. All growth indicators were measured in June, 2017. Seedling height was measured from the root collar to the apical meristem. Root collar diameter was measured 1 cm above the ground using a Vernier caliper. Leaf area was measured using a portable leaf area meter (Li-3000C, Li-COR, Lincoln, NE, USA).

Five seedlings from each replicate of each germplasm were randomly selected to measure the biomass of roots, stems and leaves after drying to constant weight at 105 °C. The variation coefficient (*CV*) of growth index was calculated as: *CV* = (standard deviation/mean) × 100%. Dickson’s quality index (QI) was calculated to quantify the quality of the seedlings: QI = SD/(HD + SR), where SD is a seedling’s total dry weight (g), HD is the ratio of a seedling’s height (mm) to root collar diameter (mm), and SR is the ratio of shoot dry weight (g) to root dry weight (g) (Aung et al., 2018).

### Photosynthetic parameters

Net photosynthetic rate (*Pn*), stomatal conductance (*Gs*), transpiration rate (*Tr*), and intercellular CO_2_ concentration (*Ci*) were measured on the third from top or younger fully-expanded leaf. Measurements were made between 9:00 and 11:00 using a portable gas-exchange system (Li-6400, Li-COR, Lincoln, NE, USA). The leaf chamber had a CO_2_ concentration of 400 μmol·mol^-1^, a light intensity of 1000 μmol·m^-2^·s^-1^, and temperature of 25°C. Plant leaves were induced with saturated light (1200 μmol·m^-2^·s^-1^) for 30 min before determining the photosynthetic parameters.

### Chlorophyll fluorescence

Chlorophyll fluorescence was measured with a portable modulating chlorophyll fluorescence meter (PAM-2000, Walz, Effeltrich, Germany) on the same functional leaves as those used to measure photosynthesis. Parameters measured included maximum fluorescence (*F*_m_^’^), steady state fluorescence (*F*_s_), and minimum fluorescence (*F*_0_^’^) in the light-adapted state. Leaves were dark adapted for 30 min before measuring initial fluorescence (*F*_o_). A single saturating radiation pulse was applied to obtain maximum fluorescence (*F*_m_). According to Genty et al. (1989), the efficiency of PSII photochemistry in the dark-adapted state was estimated by the maximum PSII photochemistry efficiency (*Fv*/*Fm*). Based on the above fluorescence parameters, the following parameters were calculated according to He et al. (2011) and Rohéček (2002). Photochemical quenching (*qP*) = (*F*_m_^’^ *- F*_s_)/(*F*_m_^’^ -*F*_o_^’^); Non-photochemical quenching (*NPQ*) =(*F*_m_ - *F*_m_^’^)/*F*_m_^’^.

### Determination of leaf chlorophyll concentration

Chlorophyll concentration was determined in the third leaf under the terminal bud of the seedling. The chlorophyll a and chlorophyll b concentrations were measured by an ethanol and acetone extraction method as described by Xu et al. (2020). The absorption of the extracts at wavelengths of 645 nm and 663 nm were measured with a Lambda 35 UV Vis spectrometer (Perkin Elmer, USA). All experiments were performed in three biological replicates and three technical replicates.

### DNA extraction, ISSR amplification and analysis

Leaves from each *P. chinensis* germplasm (five seedlings per replicate) were collected for DNA extraction. Genomic DNA was isolated using a commercial kit (Tiangen Biotech Co., Ltd., Beijing, China) according to the manufacturer’s instructions. The quality and concentration of the extracted DNA were determined by electrophoresis on 1% (w/v) agarose gels with standard lambda DNA markers as a reference. The ISSR primer set UBC100 (reference, 2006) was synthesized (Shanghai Sangon Biotechnology Co., Shanghai, China). The ten primers that amplified the highest number of fragments giving the clearest bands were selected from the set for further use (**Supplementary Table S2**). Each amplification reaction was in a final volume of 20 μL containing 1 μL (60 ng) DNA, 0.4 μL primer, 10 μL PCR MagicMix and 8.6 μL ddH_2_O. Amplification was done as follows: pre-denaturation for 5 min at 94°C, and 34 cycles of denaturation for 30 s at 94°C, 45 s at 50.5~53°C and 90 s at 72°C. The final step was a prolonged extension of 7 min at 72°C followed by storage at 4°C. The products were separated on a 1.5% (w/v) agarose gel containing ethidium bromide. Only clear, unambiguous and reproducible bands were considered for data analysis. Data was transformed into a 0/1 binary character matrix by scoring as “1” for presence and “0” for absence. The number of polymorphic bands (NPB), the percentage of polymorphic bands (PPB), effective number of alleles (Ne), Nei’s gene diversity (H) and Shannon’s information index (I) were calculated (POPGENE v. 1.32) (Yeh et al., 1997). In addition, total gene diversity (*Ht*) among populations, genetic diversity within populations (*Hs*), genetic differentiation coefficient (*G_st_*) and gene flow (*Nm*) were used to evaluate the population structure and population size. The genetic distance was calculated (POPGENE v. 1.32), imported into a numerical taxonomic and multivariate analysis system (NTSYS-pc v. 2.10) for cluster analysis and to construct a similarity matrix using the unweighted pair-group method with arithmetic mean (UPGMA) approach (Nei, 1978).

### Environmental factors

Location data, including longitude, latitude, and elevation, were obtained at the sample collection sites with a GPS (JUNO^®^ SCSD, Trimble). Climate data were obtained from the National Meteorological Data of China (http://www.escience.gov.cn/metdata/page/index.html), which provides the closest approximated values for a site based on data collected across an extensive geographical range (Sun et al., 2017). Climatic variables consisted of annual mean temperature, annual minimum and maximum temperatures, annual mean precipitation, and annual mean relative humidity (**Supplementary Table S1**).

### Statistical analysis

Data are shown as the mean ± standard deviation (SD). One-Way ANOVA and Duncan multiple comparisons (*P*<0.05 for significant differences) were used to analyze the differences of each parameters among the 15 germplasms of *P. chinensis*. Principal component analysis (PCA) was used to evaluate the all parameters and to select the best germplasms of *P. chinensis*. The above analysis was performed by SPSS software version 21.0 (IBM Inc., Chicago, IL, USA). Redundancy analysis (RDA) was performed to examine the correlations between phenotypic characteristics, genetic diversity parameters and environmental factors with the package vegan in the R v. 4.1.3 statistical environment.

## Results

### Variation in phenotypic characteristics among germplasms

There were significant differences in height, root collar diameter, leaf area, biomass, photosynthetic rate(*Pn*), stomatal conductance(*Gs*), inter-cellular CO_2_ concentration(*Ci*), transpiration rate(*Tr*), photochemical quenching coefficient(*qP*), non-photochemical quenching(*NPQ)*, chlorophyll a, and chlorophyll b among *P. chinensis* germplasms (*P*<0.05, **Supplementary Table S3**). And there was abundant variation among the phenotypic characteristics of different germplasms (**Table 1**). Leaf biomass exhibited the highest coefficient of variation (CV) at (45.49%), followed by stem biomass (36.71%), root biomass (35.92%), Chlorophyll a (35.75%), and leaf area (34.06%). Conversely, height (8.26%), *Fv*/*Fm* (3.63%) and root collar diameter (12.46%) had low CVs. Height varied approximately 30%, root collar diameter ranged more than 35%, and leaf area ranged threefold among the germplasms. Root and stem biomass varied approximately fourfold, while leaf biomass ranged more than sixfold. Overall biomass ranged nearly fourfold. The photosynthetic parameters *Pn*, *Ci*, *Tr* and *Gs* each varied about 2-fold across the 15 accessions of *P. chinensis* (**Table 1**). The value range of *Fv*/*Fm*, *qP*, and *NPQ* was 0.76–0.87, 0.14–0.44, and 0.42–0.83, respectively. Chlorophyll a concentration ranged from 0.26 to 0.81 mg·g^-1^, and chlorophyll b concentrations ranged from 0.14 to 0.43 mg·g^-1^. Germplasm NP03 demonstrated the most robust growth and photosynthetic solid capability, with the highest values for height, root collar diameter, leaf area, root biomass, stem biomass, total biomass, *Pn*, *Gs*, *qP*, chlorophyll a and chlorophyll b concentrations (**Table 1**).

**Table 1:** Variation in phenotypic characteristics among 15 germplasms of *P. chinensis*.

Phenotypic characteristics	Range	Mean	SD	CV
Height/m	17.80–23.40	20.27	1.84	8.26%
Root collar diameter/mm	3.40–4.60	4.01	0.59	12.46%
Leaf area/m^2^	10.56–29.00	17.03	2.13	34.06%
Root biomass/g	0.06–0.23	0.14	0.05	35.92%
Stem biomass/g	0.08–0.32	0.21	0.05	36.71%
Leaf biomass/g	0.10–0.61	0.29	0.02	45.49%
Total biomass/g	0.26–0.90	0.62	0.02	27.27%
*Pn*/μmol·m^-2^·s^-1^	23.46–48.76	35.57	0.27	20.35%
*Gs*/mol·m^-2^·s^-1^	0.23–0.47	0.36	0.03	21.73%
*Ci*/μmol·mol^-1^	104.76–195.02	168.71	12.09	23.15%
*Tr*/mmol·m^-2^·s^-1^	0.51–1.03	0.76	0.19	16.95%
*Fv/Fm*	0.76–0.87	0.83	0.07	3.63%
*qP*	0.14–0.44	0.27	0.13	33.95%
*NPQ*	0.42–0.83	0.63	0.19	18.55%
Chlorophyll a/mg·g^-1^	0.26–0.81	0.41	0.15	35.75%
Chlorophyll b/mg·g^-1^	0.14–0.43	0.22	0.08	33.78%

### Comprehensive analysis

**Figure 2:**
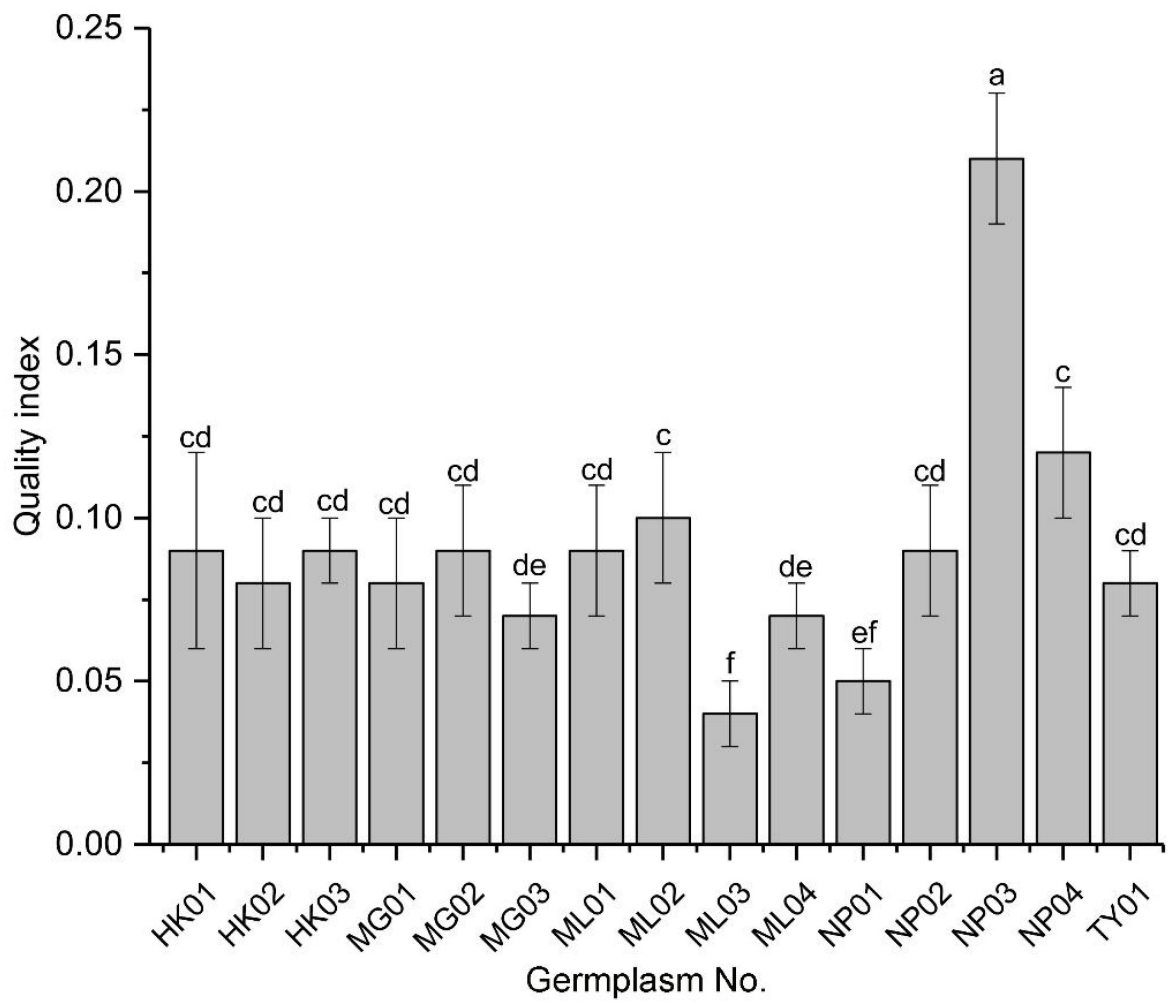
Quality index of seedlings from various *P. chinensis* germplasms. Means ± SD are shown. Different letters represent significant differences among the germplasms by ANOVA using Duncan’s multiple range test (*P* < 0.05).

The seedling quality index (*QI*) was used to synthesize the individual growth parameters and assess the overall quality of seedlings from the various germplasms. NP03 exhibited a *QI* value at least two-fold higher than any other germplasm, indicating it produced the highest quality seedlings (**Figure 2**). Among the lower *QI* values, those of ML02 and NP04 were indistinguishable but higher than those of MG03 and ML04, which were also indistinguishable from each other. Additionally, a group of eight other germplasms showed indistinguishable *QI* values among themselves and from the previous groups. Notably, ML03 and possibly NP01 had the lowest *QI* values among the germplasms tested.

In a principal component analysis of the characters collected for seedlings from the *P. chinensis* germplasms, PC1, PC2, PC3 and PC4, which had eigenvalues of 5.13, 2.37 1.87 and 1.36, respectively, accounted for 82.5% of the variance seen in the data set (**Supplementary Table S4**). The most significant principle component explained 39.425% of the variance and was mainly composed of height, *QI*, chlorophyll a and b concentrations, *Pn* and *Gs*; the contribution of PC2 was 18.198%, which reflected *Fv*/*Fm*; the contribution of PC3 was 14.354%, reflecting leaf area; and finally, the contribution of PC4 was 10.487%, which reflected *Tr*.

The original values of each trait were converted into standardized vectors. Subsequently, the scores of four PCs were obtained by substituting them into the equations. The variance contribution rate was used as the comprehensive evaluation weight to calculate the comprehensive scores for each germplasm. As shown in **Table 2**, the comprehensive score for NP03 was significantly higher than for the other germplasms. Based on the comprehensive scores, the germplasms in the top 20% were screened, including NP03, NP01, and ML02.

**Table 2:** The score of principal component analysis of *P. chinensis*.

Germplasm	The score of PC1	The score of PC2	The score of PC3	The score of PC4	Composite	Order
HK01	-0.704	0.427	1.214	-0.277	0.661	7
HK02	-0.482	-0.108	-1.290	1.383	-0.498	9
HK03	1.871	-0.439	-0.805	-0.778	-0.150	8
MG01	-0.184	-1.870	-0.896	0.982	-1.968	11
MG02	-0.702	-3.510	-0.574	0.438	-4.349	15
MG03	0.576	-0.293	-1.275	-2.014	-3.006	12
ML01	-1.875	-1.605	1.586	-1.517	-3.411	13
ML02	-0.495	-0.658	2.497	1.697	3.041	3
ML03	-2.280	0.731	-1.555	-0.889	-3.992	14
ML04	-3.132	0.706	1.390	-0.285	-1.321	10
NP01	-0.217	2.512	0.748	0.120	3.162	2
NP02	2.087	0.746	-0.756	-1.017	1.061	5
NP03	6.394	0.064	1.515	-0.038	7.935	1
NP04	0.633	1.160	-1.746	2.033	2.079	4
TY01	-1.489	2.137	-0.053	0.162	0.757	6

**Figure 3:**
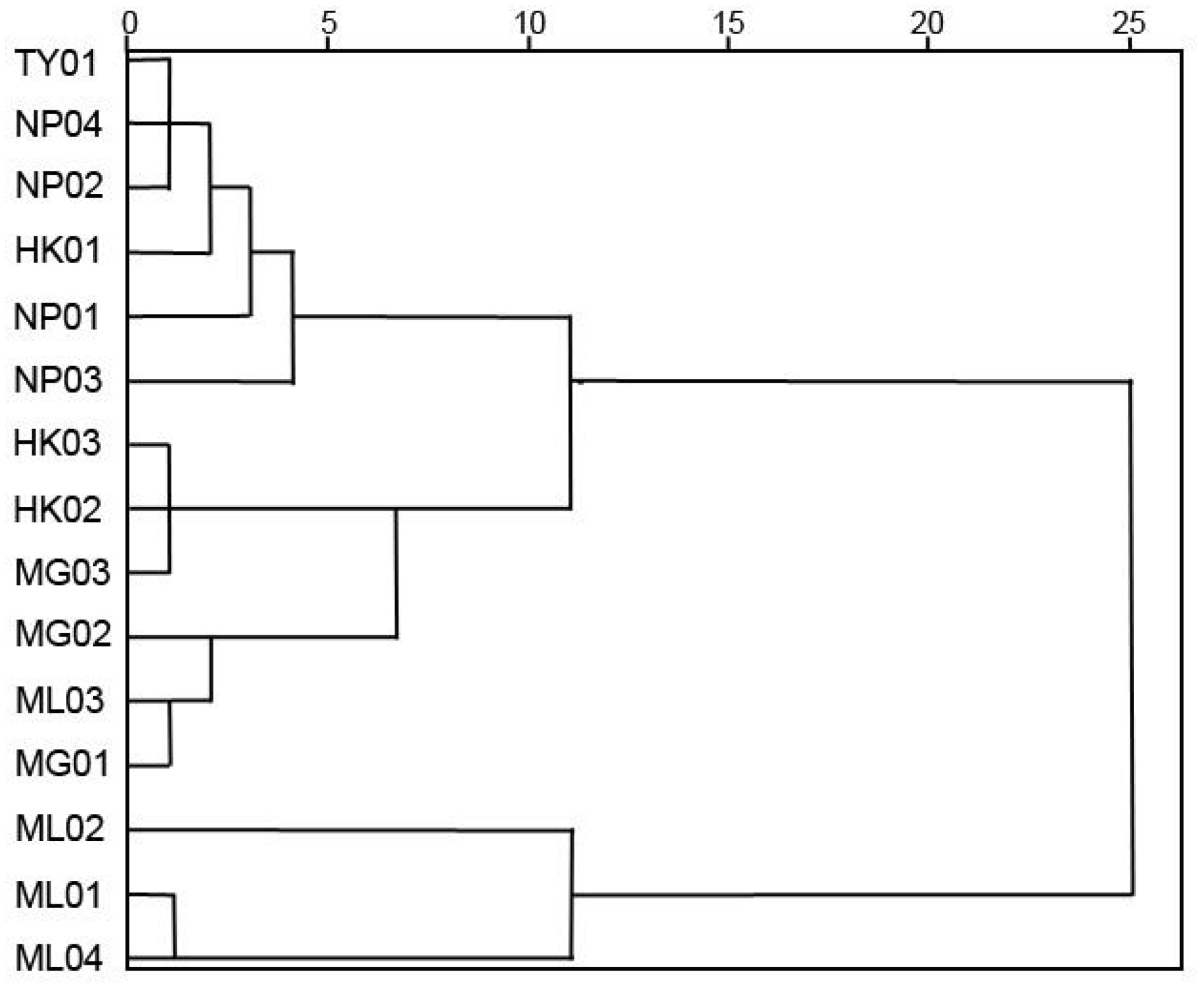
Cluster diagram of 15 P*. chinensis* germplasms based on quantitative traits.

According to Ward’s clustering analysis, the 15 P*. chinensis* germplasms were clustered into 5 clusters at 6 Euclidean distances (**Figure 3**). Group I comprised six germplasms, namely NP01, NP02, NP03, NP04, TY01, and HK01, mainly from Guangxi Province. Group II consisted of three germplasms, including HK02, HK03, and MG03, originating from Hekou and Maguan counties of Yunnan Province. Group III included germplasms MG01, MG02, and ML03. Furthermore, Group IV included only one germplasm, ML02, while Group V included two germplasms, ML01 and ML04 (**Figure 3**).

### Genetic diversity analysis based on ISSR molecular markers of *P. chinensis*

A set of 100 ISSR primers were used to evaluate polymorphisms in the 15 P*. chinensis* germplasms. Ten primers were selected for the complete analysis based on polymorphism detection, reproducibility and readability (**Supplementary Table S2**). Using these 10 primers, a total of 82 bands were amplified within the 15 germplasms. Among them, 50 amplified bands were polymorphic, resulting in an average polymorphic band percentage of 63.83%. The PPB obtained by amplification of the UBC880 primer was 41.67%, while the PPB obtained from amplification of the other nine primers exceeded 50%. Therefore, the selected ten ISSR primers exhibited significant amplification polymorphism and could effectively identify the genetic diversity of *P. chinensis*.

POPGEN 1.32 software was used to calculate the genetic diversity index of 15 germplasms. The main genetic parameters are reported in **Table 3**. Following ISSR analysis, the PPB was 61.36%, the Ne was 1.2887, the H was 0.1879 and the I was 0.3258 at the species level. At the population level, the values for each index varied across the germplasms. PPB ranged from 8.13% for the ML03 to 26.85% for the NP03, with a mean value of 16.58%. The Ne varied from 1.1013 for the HK03 to 1.2978 for the NP03, with a mean value of 1.1609. The Nei’s gene diversity (*H*) ranged from 0.0612 for the ML03 to 0.1128 for the NP03, with a mean value of 0.0801. Additionally, the Shannon diversity index (*I*) ranged from 0.1002 for the ML03 to 0.2213 for the NP03, with an average of 0.1356. The *I* of six accessions were higher than average, including HK03, MG03, ML02, ML04, NP02, and NP03, which showed that these *P. chinensis* materials had rich genetic diversity. NP03 demonstrated the highest genetic diversity (PPB=26.85%, H=0.1128, I=0.2213), while ML03 exhibited the lowest genetic diversity (PPB=8.13%, H=0.0612, I=0.1002).

**Table 3:** Genetic diversity of 15 germplasms by ISSR markers.

Germplasm	Percentage of polymorphic bands (PPB)/%	Effective allele number (*Ne*)	Nei’s gene diversity (H)	Shannon diversity index (I)
HK01	8.75	1.1112	0.0658	0.1029
HK02	13.89	1.1523	0.0759	0.1221
HK03	26.3	1.1013	0.1012	0.1656
MG01	9.6	1.1198	0.0679	0.1089
MG02	12.41	1.1112	0.0689	0.1138
MG03	26.11	1.1887	0.0911	0.1614
ML01	10.88	1.1298	0.0699	0.1109
ML02	19.87	1.1865	0.0878	0.1542
ML03	8.13	1.2011	0.0612	0.1002
ML04	16.58	1.1734	0.0812	0.1426
NP01	16.49	1.1652	0.0789	0.1321
NP02	26.12	1.1996	0.0915	0.1642
NP03	26.85	1.2978	0.1128	0.2213
NP04	12.87	1.1329	0.0738	0.1154
TY01	13.78	1.1426	0.0743	0.1189
Mean	16.58	1.1609	0.0801	0.1356
Species level	61.36	1.2887	0.1879	0.3258

Genetic differentiation analysis of the 15 germplasms revealed that the Ht was 0.1876, Hs was 0.0864, and *Gst* was 0.6264 (>0.5) (**Table 4**). This indicates that 62.64% of the total genetic variation occurred among populations, while 37.36% occurred within populations, indicating high genetic differentiation. Additionally, *Nm* was calculated as 0.3736 (<1) (**Table 4**), suggesting weak gene flow among populations and significant genetic differentiation among populations of *P. chinensis* is prominent.

**Table 4:** Genetic differentiation among 15 germplasms of *P. chinensis*.

All population	Total gene diversity (*Ht*)	Genetic diversity within populations (*Hs*)	Genetic differentiation coefficient (*G_st_*)	Gene flow (*Nm*)
Mean	0.1876	0.0864	0.6264	0.3736
St. Dev	0.0241	0.0106		

**Figure 4:**
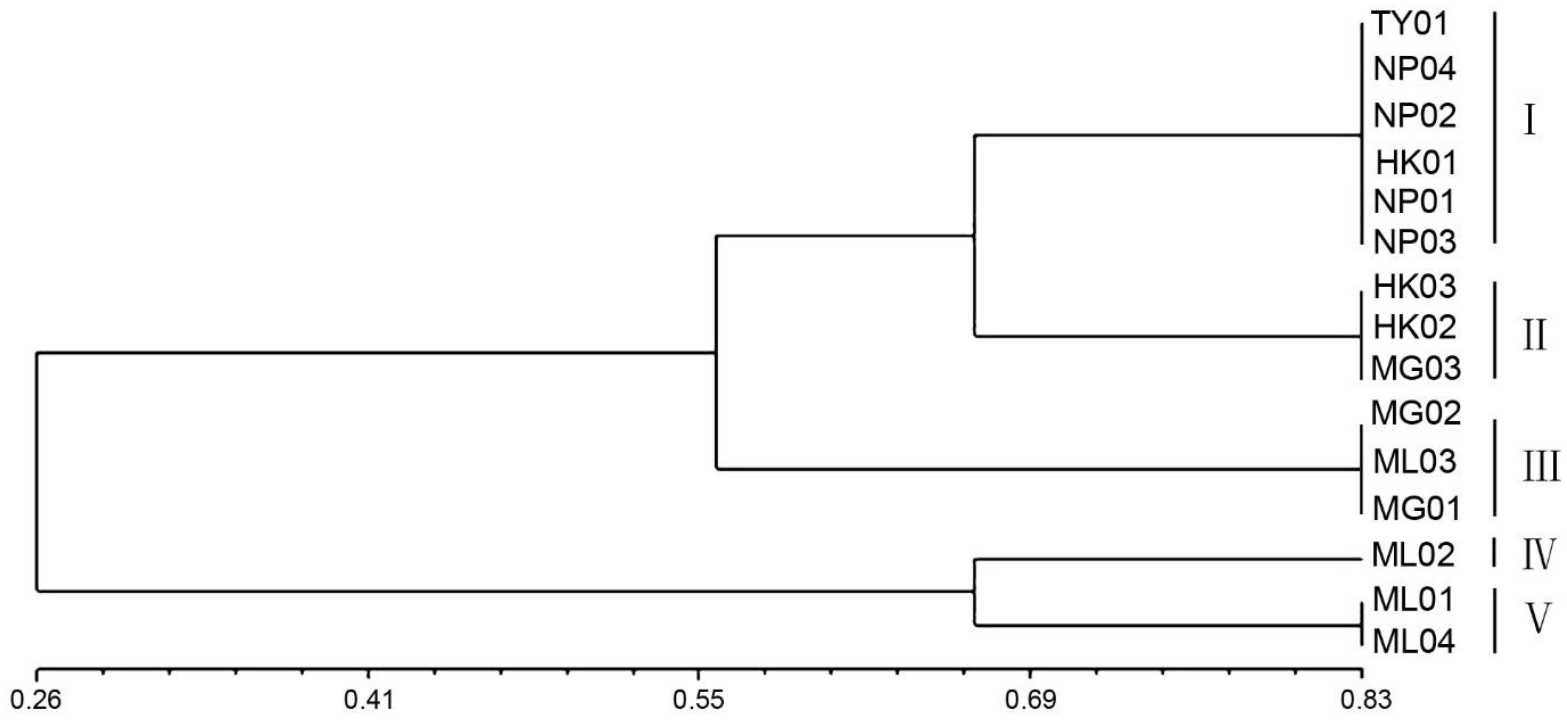
The phylogenetic dendrogram for the 15 germplasms of *P. chinensis* based on genetic distances.

NTSYS-pc (V2.10) software was utilized for cluster analysis using the UPGMA method, resulting in a phylogenetic dendrogram of 15 germplasms of *P. chinensis* (**Figure 4**). The genetic similarity coefficients of 15 P*. chinensis* germplasms ranged from 0.55 to 0.66, indicating ich genetic diversity within these germplasms. Furthermore, the germplasms were clustered into five groups, with a similarity coefficient of 0.66 used as the segmentation criterion. Comparing the clustering results in **Figure 3** and **Figure 4**, the results obtained by the two clustering methods were basically similar. Both methods successfully grouped germplasms from the same area into the same cluster. For example, the germplasms HK01, NP01, NP02, NP03, NP04, and TY01 were clustered in group I, suggesting a close genetic relationship among germplasms from Guangxi Province. Similarly, HK02, HK03, and MG03 germplasms clustered in group II, while MG01, MG02, and ML03 were clustered in group III. Additionally, ML02 clustered in group IV, and ML01 and ML04 clustered in group V.

### The relationship between phenotypic characteristics, genetic diversity and environmental factors

**Figure 5:**
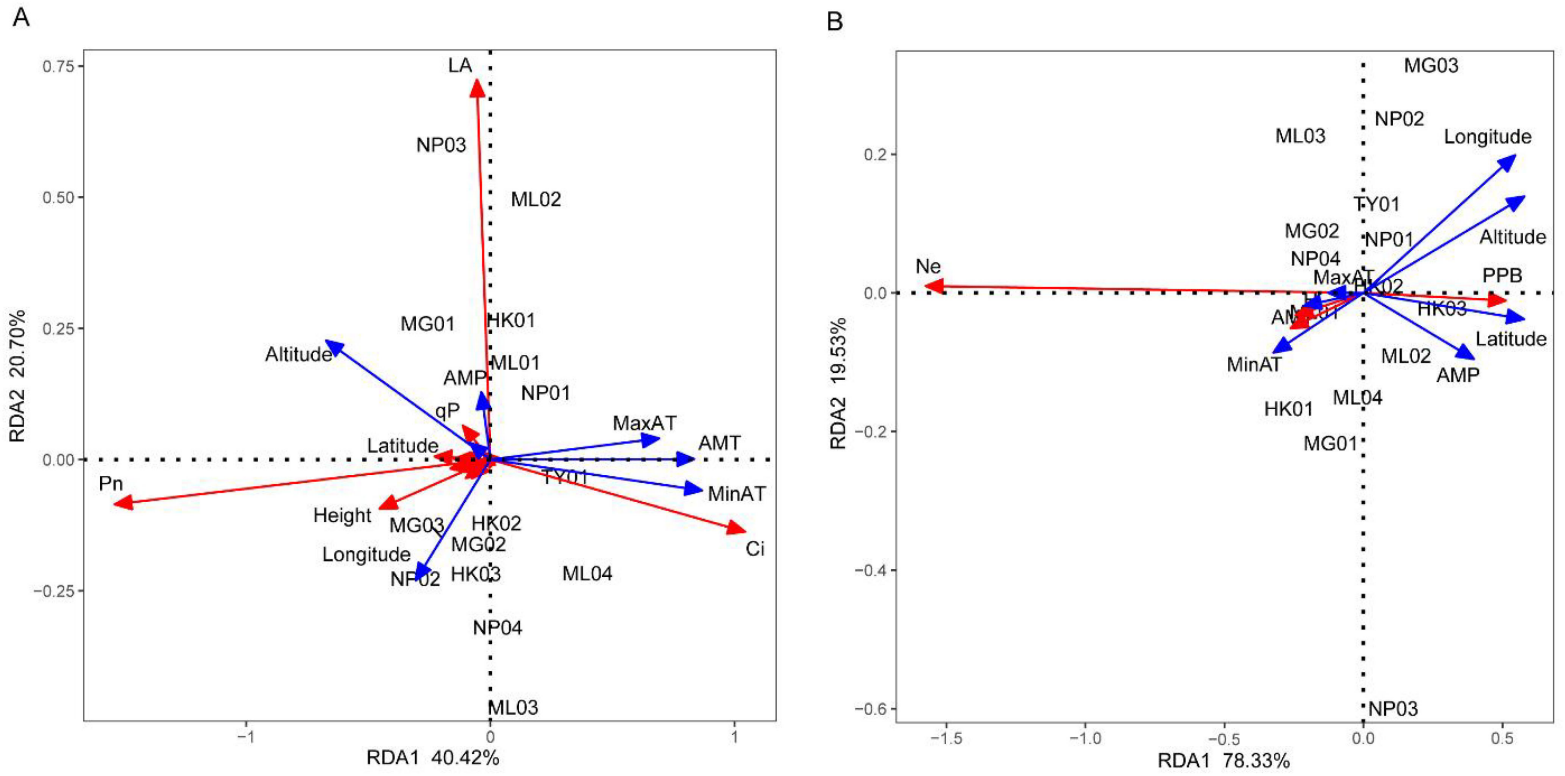
Redundancy analysis of the relationship between phenotypic characteristics **(A)**, genetic diversity **(B)** and environmental factors. RCD, LA, RB, SB, LB, TB, QI, ChlA, ChlB, PPB, Ne, H, I, AMT, MinAT, MaxAT, AMP, AMRH stand for root collar diameter, leaf area, root biomass, stem biomass, leaf biomass, total biomass, Quality index, Chlorophyll a, Chlorophyll b, percentage of polymorphic bands, effective allele number, Nei’s gene diversity, Shannon diversity index, annual mean temperature, minimum air temperature, maximum air temperature, annual mean precipitation, and annual mean relative humidity, respectively. The red arrows represent phenotypic characteristics **(A)** and genetic diversity parameters **(B)** respectively, while the blue arrows represent environmental factors.

RDA was used to test whether environmental factors influence the phenotypic characteristics and genetic diversity of *P. chinensis*. As shown in **Figures 5A, B**, the cumulative explanatory variable of environmental factors for phenotypic traits and genetic diversity were 61.12% and 97.86%, respectively. This suggests that the first two axes capture most of the information regarding the relationship between phenotypic characteristics, genetic diversity and environmental factors. In the RDA sorting diagram, the angle between the arrow projection on the sorting axis and the arrow line represents the correlation. An acute angle indicates a positive correlation, while an obtuse angle indicates a negative correlation. Moreover, a smaller angle signifies a higher correlation. In **Figure 5A**, altitude, longitude, maximum air temperature, annual mean temperature and minimum air temperature were the top five factors contributing to the RDA model. Phenotypic traits such as Pn and height showed a significant positive correlation with altitude and longitude. Ci exhibited a significant positive correlation with the maximum air temperature, annual mean temperature and minimum air temperature, while leaf area displayed a close positive correlation with altitude and annual mean precipitation. In **Figure 5B**, altitude, longitude, latitude, annual mean precipitation and minimum air temperature were the top five factors contributing to the RDA model. Ne, H and I were closely and positively correlated with the annual mean, minimum, and maximum air temperatures. Additionally, PPB positively correlated with altitude, longitude, latitude and annual mean temperature.

## Discussion

### Phenotypic variation and its relationship with environmental factors of *P. chinensis*

Phenotypic traits are the comprehensive effect of plant genotype and environmental factors and are an essential embodiment of genetic variation, which can directly reflect the richness of species gene resources. Trait variation is a prerequisite for the formation of plant varieties. The coefficient of variation reflects the plasticity of a trait on the one hand and the stability of the trait on the other. We found that 15 germplasms from Guangxi and Yunnan provinces had significant differences in seedling height, root collar diameter, leaf area, biomass, photosynthetic parameters, chlorophyll fluorescence parameters and chlorophyll content. In the diversity analysis, the variation coefficient can reflect the dispersion degree of the trait to a certain extent. The larger the variation coefficient, the higher the dispersion degree (Xin et al., 2022). Generally, the variation coefficient is greater than 10%, indicating a significant difference between different germplasms of this trait (Xin et al., 2022). The variation coefficient of the phenotypic traits of *P. chinensis* is between 3.63% and 45.49%. Except that the variation coefficient of Height (8.26%) and Fv/Fm (3.63%) is less than 10%, the variation coefficient of the other 14 traits is more than 10%. This suggests that the growth characteristics of different germplasms of *P. chinensis* are diverse, and excellent germplasm resources can be screened from them.

Environmental factors are the key constraints affecting plant distribution and growth on a large geographical scale. The differences in temperature, precipitation and other factors in different locations lead to significant differences in the growth adaptability of different provenances to their habitats (Yuan et al., 2021). Studies have shown that the height, root collar diameter and biomass of *L. formosana* and *P. bournei* from different provenances are closely related to geographical and climatic factors such as altitude and temperature (He et al., 2019; Tan et al., 2019). This study also drew a similar conclusion that altitude, longitude, maximum air temperature, annual mean temperature and minimum air temperature were the main environmental factors that affected the variation of phenotypic characteristics of *P. chinensis*. It is speculated that *P. chinensis* can adapt to climate change, especially temperature change, by changing its phenotypic traits.

### Genetic diversity and its relationship with environmental factors of *P. chinensis*

The genetic diversity of rare and endangered plants with limited geographical distribution is generally believed to be low. However, some recent studies have found that rare and endangered plants also have high genetic diversity. The low number does not necessarily mean their genetic diversity is low (Schwartz, 1985). For example, Jin and Li (2007) reported that the endangered plant *Calycanthus chinensis* W. C. Cheng & S. Y. Chang had high genetic diversity at the species level but low genetic diversity at the population level. Our study came to a similar conclusion, that is, at the species level, the PPB was 61.36%, the Ne was 1.2887, H was 0.1879, and I was 0.3258; this reveals that the 15 accessions of *P. chinensis* exhibit rich genetic diversity. However, at the population level, the PPB, Ne, H, and I were 16.58%, 1.1609, 0.0801, and 0.1356, respectively, indicating relatively low genetic diversity. The causes of gene decline within populations may be related to genetic drift, decline in adequate size and self-compatibility (Zou et al., 2001).

Gene differentiation and gene flow are critical indicators to evaluate the genetic structure of species. In our study, following analysis of the ISSR marker, we observed that the Ht value in *P. chinensis* was 0.1876, which was lower than the general trend for long-lived woody perennial species (Ht=0.28 from 195 entries) (Hamrick et al., 1992). The Gst was 0.6264. That is, 62.64% of the total genetic variation occurred among the populations and 37.36% occurred within the populations, suggesting that variation is more prevalent among populations. The germplasm resources came from Mengla, Hekou, Maguan, Napo and Tianyang counties, all belonging to alpine landforms. The distance between the mountains blocked the gene flow, which led to the increase of inbreeding and genetic drift within the local population, resulting in the decrease of genetic diversity within the population and large genetic differentiation among the populations (García-Verdugo et al., 2019, 2015; Jaros et al., 2018). Gene flow refers to the gene movement within and between populations, which is negatively correlated with gene differentiation and is extremely important for population transfer and plant evolution (Slarkin, 2003). Wright (1931) pointed out that genetic differentiation between populations plays a role in homogenization only when the value of Nm is more significant than one. If the value of Nm is less than 1, it indicates that gene flow is restricted, leading to increased genetic differentiation between populations. In the current study, the Nm was 0.3736, which showed great genetic differentiation between *P. chinensi*s populations. It was speculated to be related to the growth environment and biological characteristics of *P. chinensis*. As a rare and endangered plant with high economic value, *P. chinensis* is seriously affected by human disturbance and destruction, which directly leads to a sharp decrease in the number of individuals in the population, serious population fragmentation and intensified genetic drift. In addition, the narrow distribution regions often growing in the virgin ravine rainforest and mountain rainforest and the limited gene flow caused by geographical isolation may be one of the reasons for the high genetic differentiation among populations and the low level of genetic diversity. Endangerment can lead to a decrease in the level of genetic diversity, and then low levels of genetic diversity can make it more endangered.

Genetic diversity reflects the characteristics of species in origin, evolution and environmental adaptation (Li et al., 2008; Szczecińska et al., 2016). The diversity of the population is produced by adapting to environmental changes in the process of evolution. The diversity level can reflect species’ evolutionary potential (Frankel and Soulé, 1981). Species with rich genetic diversity have more alleles, better adaptability, and stronger resistance to environmental changes (Li et al., 2018). In our study, two methods, phenotypic traits variation analysis and ISSR molecular markers, were used to verify the great differences in the genetic background of different germplasm of *P. chinensis*. 10 pairs of ISSR primers were selected to analyze the genetic diversity of 15 germplasms of *P. chinensis*. The results showed that 83 loci were amplified using the ten primer pairs. Of these loci, 50 resulted in polymorphic bands and the polymorphism rate was 63.83%; on average, five polymorphic loci were amplified per primer pair. The ISSR marker is highly polymorphic, suggesting that the natural forest of *P. chinensi*s has a very rich gene polymorphism.

Additionally, the results revealed that the percentage of polymorphic loci of the 15 germplasms varied from 8.13% (ML03) to 26.85% (NP03), and the average percentage of polymorphic loci for the 15 germplasms was 16.58%. This suggests that NP03 had high genetic diversity and high adaptability. In general, the greater the genetic diversity of a species, the greater its resilience to environmental change. The difference of genetic structure among populations of plants can improve their ability to resist adverse environments, expand their distribution range, and enhance their adaptability. Scholars have studied the correlation between environmental factors and the genetic diversity of different plant populations and found that there is a specific correlation between population genetic diversity and factors such as altitude, temperature and precipitation (Nybom, 2004; Turpeinen et al., 2001), indicating that environmental factors lead to gradient changes in spectral band frequency and genetic diversity to a certain extent, resulting in corresponding adaptive ecogeographic differentiation (Eckert et al., 2010; Howe et al., 2003). The results showed that annual mean precipitation and minimum temperature were significant factors affecting genetic diversity, indicating that these environmental factors may affect the expression of stress-related genes, and ISSR polymorphisms in *P. chinensis* produce selection pressure. In addition, the phenotype of the tree may be changed by regulating the expression of related genes, so as to adapt to different environments. This also confirms from the side that as a tropical rain forest tree species, it is sensitive to precipitation and temperature, extremely strict to habitat requirements, and only grows in hot and humid areas. Furthermore, global warming may also have an impact on the distribution or adaptation mechanisms of the tree. In conclusion, the causes of genetic differentiation among populations of *P. chinensis* are not only related to natural selection and lack of effective gene flow among populations, but also related to biological characteristics and other causes, so the correlation between genetic differentiation, natural selection and biological characteristics needs to be further studied.

### Geographical variation of genetic diversity of *P. chinensis*

The results of this study showed that climatic factors such as temperature, altitude and precipitation were important environmental factors affecting the genetic variation of *P. chinensis* natural forest, and habitats with different latitude and longitude was the difference in their climatic conditions. Correlation with climatic factors of producing areas meant that there was a certain geographical pattern, which further indicated that the geographical variation of gene level of *P. chinensis* population was not completely random, but has a certain regularity. The cluster analysis based on phenotypic traits and genetic diversity showed that the natural forests of *P. chinensis* distributed in Guangxi had high similarity, while the natural forests distributed in other regions had certain independent genetic characteristics. Shannon diversity index (I) and Percentage of polymorphic bands (PPB) can be used to evaluate genetic diversity. The higher the index is, the higher the genetic diversity is. The I and PPB of HK03 and NP03 were high, suggesting that these two populations distributed in Guangxi had relatively frequent communication with other populations, and the level of gene polymorphism was high. The populations of Hekou, Maguan and Mengla were relatively independent, indicating that the environment of these populations limited the communication with other populations, and their genetic characteristics were unique, with low I and PPB. This further indicates that there is a certain geographical variation pattern in the genetic variation of *P. chinensis* natural population.

### Evaluation of excellent germplasm of *P. chinensis*

Due to the instability of phenotypic traits, it is difficult for a single index to accurately and comprehensively reflect the growth of plants. The combination of phenotypic indexes and genetic relationships obtained by molecular markers to screen excellent germplasm can enhance the reliability of screening results and draw more scientific conclusions. QI values were calculated based on seven growth indexes, and the results showed that the top 3 germplasm were NP03, NP04 and ML02. Principal component analysis was performed on a total of 17 indexes, and it was found that the top 3 germplasm with principal component scores were NP03, NP01, and ML02. Two germplasm, NP03 and ML02, were screened simultaneously by the above two methods. Meanwhile, the results of cluster analysis of phenotypic traits and ISSR markers showed that NP03 and ML02 existed in different groups, indicating that NP03 and ML02 were superior germplasm resources among the 15 germplasm. Principal component analysis was performed on 17 indicators in this study, and the first 4 principal components represented 83.85% of the information of the original data, which were closely related to height, QI, chlorophyll a, chlorophyll b, Pn, Gs, Fv/Fm, leaf area, and Tr. The coefficient of variation of leaf area (34.06%) and chlorophyll content (33.78-35.75%) are large, and the determination method are relatively simple. Leaves are the main photosynthetic organs of higher plants and play an important role in plant physiological functions (Li and Tian, 2022). Leaf area, as an important indicator of plant functional traits, has been widely used to evaluate ecological adaptability and carbon assimilation ability of plants (Bajwa et al., 2021; Wong and Gamon, 2015). Chlorophyll is vital for photosynthesis, which allows plants to absorb energy from light (Xu et al., 2020). In addition, leaf phenotypic traits directly affect some physiological activities of plants and the ability of plants to utilize resources, which reflects the survival adaptation strategy adopted by plants to obtain the maximum carbon assimilation (McCulloh and Sperry, 2005). Leaf phenotypic variation is also a common reflection of plant genetic variation and environmental interaction. In conclusion, leaf area and chlorophyll content can be used as the main evaluation indexes, which can provide the basis for the screening and breeding of high-quality provenances of *P. chinensis*.

Based on the analysis of germplasm diversity of 15 germplasms, the results showed that there were rich morphological and genetic diversity in Guangxi and Yunnan province, which provided basic germplasm sources for the mining of excellent germplasm of *P. chinensis*. Simultaneously, the high degree of genetic differentiation and low level of genetic diversity among populations were one of the main reasons for the endangerment of *P. chinensis*. The genetic variation of the natural population of *P. chinensis* had a certain geographical variation pattern. In the future, the standardized management, *in situ* conservation and *ex situ* conservation should be strengthened based on the growth characteristics and genetic diversity of *P. chinensis*.

## Data Availability

The original contributions presented in the study are included in the article/**Supplementary Material**. Further inquiries can be directed to the corresponding author.

## Supplementary material

Supplementary Table S1 Geographic locations and climatic conditions of the 15 germplasms used in this study.

Supplementary Table S2 Inter simple sequence repeat (ISSR) primers and amplification results.

Supplementary Table S3 Phenotypic characteristics among 15 germplasms of *Parashorea chinensis*.

Supplementary Table S4 Character value and contribution rate of principal components.
